# More extensive hypometabolism and higher mortality risk in patients with right- than left-predominant neurodegeneration of the anterior temporal lobe

**DOI:** 10.1186/s13195-022-01146-w

**Published:** 2023-01-10

**Authors:** Lars Frings, Ganna Blazhenets, Raphael Binder, Tobias Bormann, Sabine Hellwig, Philipp T. Meyer

**Affiliations:** 1 Department of Nuclear Medicine, Medical Center - University of Freiburg and Faculty of Medicine, University of Freiburg, Freiburg, Germany; 2 Center of Geriatrics and Gerontology, Medical Center - University of Freiburg and Faculty of Medicine, University of Freiburg, Freiburg, Germany; 3 Department of Neurology and Clinical Neuroscience, Medical Center - University of Freiburg and Faculty of Medicine, University of Freiburg, Freiburg, Germany; 4 Department of Psychiatry and Psychotherapy, Medical Center – University of Freiburg and Faculty of Medicine, University of Freiburg, Freiburg, Germany

**Keywords:** Frontotemporal dementia, Semantic dementia, rtvFTD, Anterior temporal lobe, FDG PET, Survival

## Abstract

**Background:**

Left-predominant neurodegeneration of the anterior temporal lobe (ATL) and the associated syndrome termed semantic variant primary progressive aphasia (svPPA) are well characterized. Less is known about right-predominant neurodegeneration of the ATL, which has been associated with the clinical syndrome named right temporal variant of frontotemporal dementia (rtvFTD). Here, we assessed glucose metabolism across the brain, cognitive performance, and mortality in patients with right-predominant neurodegeneration of the ATL.

**Methods:**

Patients with predominant hypometabolism of the ATL on FDG PET (as a measure of neurodegeneration) were retrospectively identified and categorized into those with asymmetrical right, left, or symmetric bilateral involvement (*N* = 10, 17, and 8). We compared whole-brain, normalized regional glucose metabolism using SPM12, cognitive performance on the CERAD Neuropsychological Assessment Battery, and mortality risk (age- and sex-adjusted Cox proportional hazard model) between groups.

**Results:**

Hypometabolism was most pronounced and extensive in patients with right-predominant neurodegeneration of the ATL. Beyond the right temporal lobe, right frontal and left temporal lobes were affected in these patients. Cognitive performance was similarly impaired in all three groups, with predominant naming and hippocampal-dependent memory deficits. Mortality risk was 6.1 times higher in patients with right- than left-predominant ATL neurodegeneration (*p* < 0.05). Median survival duration after PET was shortest in patients with right- and longest in patients with left-predominant ATL neurodegeneration (5.7 vs 8.3 years after examination).

**Discussion:**

More extensive neurodegeneration and shorter survival duration in patients with right- than left-predominant neurodegeneration of the ATL might indicate that the former consult memory clinics at a later disease stage, when symptoms like naming and episodic memory deficits have already emerged. At the time of diagnosis, the shorter survival duration of patients with right- than left-predominant ATL neurodegeneration should be kept in mind when counseling patients and caregivers.

**Supplementary Information:**

The online version contains supplementary material available at 10.1186/s13195-022-01146-w.

## Background

Focal neurodegeneration of the anterior temporal lobe (ATL) is most frequently due to fronto-temporal lobar degeneration (FTLD), often leading to frontotemporal dementia (FTD). The type of symptoms patients develop depends on the predominantly affected hemisphere: Left-predominant ATL degeneration is associated with impaired naming and single-word comprehension and hence the clinical syndrome of semantic variant primary progressive aphasia (svPPA) or “left semantic dementia (SD)”. The clinical syndrome of svPPA has been very well characterized [1]. By contrast, much less is known about the right-hemisphere variant of ATL neurodegeneration: The main symptoms reported in previous studies were either behavioral alterations [2, 3], prosopagnosia/problems with recognition of famous faces [4], or both [5, 6]. The associated syndrome has been termed right SD [7] or right temporal variant of FTD (rtvFTD) [6].

Even less is known about the survival duration and mortality risk of patients with right-predominant neurodegeneration of the ATL. For SD and svPPA, average survival durations of 8 [8], 11 [9], or 12 years [10, 11] after symptom onset and 3 [8] or 9 years [9] after diagnosis have been reported. Only one study so far reported survival in right-predominant ATL neurodegeneration patients [12]: The authors investigated SD patients and reported a median survival of 12 years after symptom onset for SD with right-predominant (*N* = 24) and 14 years for SD with left-predominant atrophy of the ATL on MRI (*N* = 70); survival durations did not differ significantly. In this study, lacking information hampered the interpretability of the survival data (e.g., it was not reported whether age- or sex-adjusted survival was different between the left and right groups). To our knowledge, no other studies assessing survival duration or mortality risk in right ATL neurodegeneration exist, rendering the data basis for patient counseling in clinical practice weak.

Notably, most of the above-cited previous studies included only patients with a specific clinical syndrome (most often SD), which impairs the identification of atypical clinical symptoms. For example, whereas “preservation of day-to-day memory” was among the consensus criteria for SD [13], studies which investigated patients with ATL neurodegeneration (not restricted to SD) also found episodic memory deficits [5, 6]. Here, we aimed to characterize patients with right ATL neurodegeneration and any cognitive impairment, irrespective of the presence of specific syndromes like SD.

We identified and characterized patients with right-predominant neurodegeneration of the ATL regarding (1) hypometabolism across the brain, (2) type and extent of cognitive deficits, and (3) mortality risk. We compared them to patients with left-predominant and patients with symmetric neurodegeneration of the ATL.

## Methods

### Patients

**Fig. 1:**
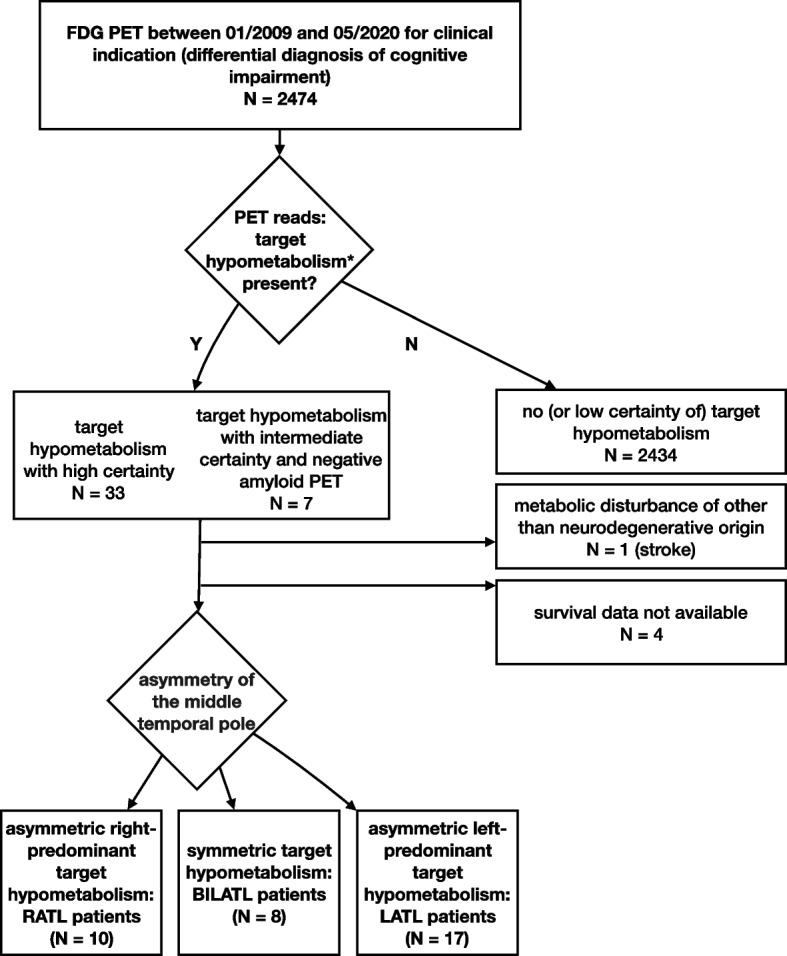
Schematic display of patient selection and assignment to analyzed groups. RATL, right-predominant, LATL, left-predominant, BILATL, symmetric neurodegeneration of the ATL. *The target hypometabolism, a metabolic pattern for svPPA or SD, was defined as predominant hypometabolism of the left, right, or both ATL. This hypometabolism may extend to frontal and (inferior) parietal cortices, but with a clear-cut gradient from the temporal pole to other regions and without clearly suggesting other neurodegenerative syndromes or diseases (e.g., Alzheimer’s disease, behavioral variant FTD, dementia with Lewy bodies, or atypical parkinsonian syndromes)

We included patients conforming with the following inclusion and exclusion criteria (Fig. 1).

Cerebral [^18^F]fluorodeoxyglucose (FDG) PET was carried out at our institution for differential diagnosis of cognitive impairment between 01/2009 and 05/2020 (inclusion criterion 1). These were searched by an experienced clinician (P.T.M.; > 20 years of neuroimaging experience with PET and SPECT) for a metabolic pattern typical for svPPA or SD (inclusion criterion 2), which was defined as predominant hypometabolism of the left, right, or both ATL. This hypometabolism may extend to frontal and (inferior) parietal cortices, but with a clear-cut gradient from the temporal pole to other regions and without clearly suggesting other neurodegenerative diseases (e.g., Alzheimer’s disease, behavioral variant of FTD (bvFTD), dementia with Lewy bodies, or atypical parkinsonian syndromes). FDG PET were read in a highly standardized fashion based on five by six axial slice displays (scaled to brain parenchyma uptake and using an identical hot-metal colorscale and thresholding) and NEUROSTAT 3D-SSP decrease displays compared to age-matched controls [14]. On a 4-point-scale, the rater marked whether a metabolic pattern typical for svPPA or SD was present (not present or present with low, intermediate, or high certainty). We included patients with high certainty of svPPA or SD metabolic pattern and patients with intermediate certainty plus a negative amyloid PET (inclusion criterion 3). PET reads were blind to all clinical details, e.g., type of cognitive deficits. Patients with metabolic disturbance of other than neurodegenerative origin and those without available survival data were excluded (exclusion criteria 1 and 2).

Notably, in contrast to several previous studies, we did not use a clinical syndrome (e.g., SD, svPPA, or bvFTD) as an inclusion criterion in order to be able to characterize the biological entity of right ATL neurodegeneration.

The institutional Ethics Committee approved this retrospective study (application No. 351/16). All patients gave written informed consent to retrospective analysis of their data.

### FDG PET acquisition and processing

PET images were acquired on a Siemens ECAT PET scanner (40–60 min p.i.; *N* = 9), Philips Gemini TF64 (*N* = 21), or Philips Vereos (*N* = 5) PET/CT systems (50–60 min p.i.) after injection of 292 ± 29, 219 ± 31, or 213 ± 7 MBq FDG, respectively. For details on data acquisition and reconstruction, please see [15, 16]. In order to account for different spatial resolutions of the scanners, reconstructed and spatially normalized images were smoothed with Gaussian filters of 5 (ECAT), 7 (TF64), or 8 mm FWHM (Vereos) using SPM12 (Version 7219; www.fil.ion.ucl.ac.uk). After scaling to individual brain parenchyma uptake, PET images were subjected to statistical analyses with SPM12.

### Cognitive assessment

The German version of the Consortium to Establish a Registry for Alzheimer’s Disease Neuropsychological Assessment Battery (CERAD-NAB [17]) was used as part of the routine clinical work-up in a memory clinic setting. Analyzed test scores were semantic fluency (animals), Boston Naming Test (number of correct responses), word list learning (sum of three trials), word list recall (absolute number of recalled words and relative recall, i.e., percent of words recalled in the third learning trial), word list recognition, figure drawing, and figure recall (absolute and percent of previously drawn). Raw test scores were converted to age-, sex-, and education-corrected *z* scores with reference to normative test data [18].

### Survival data

Survival data of individual patients were acquired by automated queries of the state resident registry in 08/2021 or from clinical records of the hospital information system, yielding either confirmation of survival or the date of death. For the main survival analysis, we used survival durations from the time of FDG PET. We only secondarily used survival durations from symptom onset, as the latter is commonly only a rough estimate in retrospect.

### Control group data

For the definition of symmetric ATL metabolism and for comparison of regional FDG uptake, we used FDG PET data from 35 healthy elderly persons and 45 age-matched control patients scanned under identical conditions from our database for statistical comparisons, in whom somatic CNS diseases were carefully excluded. Controls were imaged on Philips TF64 (*N* = 35) or Philips Vereos (*N* = 45) PET/CT systems (Table 1).

**Table 1:** Description of patients and controls

	RATL patients	LATL patients	BILATL patients	Control subjects
*N*	10	17	8	80
Age (mean ± S.D.)	66.4 ± 6.9	68.7 ± 5.7	68.0 ± 6.5	69.4 ± 10.7
Sex (female to male)	4:6	12:5	4:4	39:41
Education *	12.6 ± 2.9	14.0 ± 2.9	12.9 ± 3.3	N.A
Symptom duration, years (mean ± S.D.)**	2.8 ± 1.6	3.2 ± 2.2	3.0 ± 1.8	N.A
MMSE score **	23.4 ± 5.4	23.3 ± 3.7	23.8 ± 1.8	N.A
Amyloid PET (Proportion of negative from all available cases)	5/5	8/8	6/6	N.A
Predominant clinical symptoms	Behavioral: 8 Language: 1 Other: 1	Behavioral: 2 Language: 10 Memory: 5	Behavioral: 2 Language: 3 Memory: 3	N.A

*RATL*, Right-predominant, *LATL* Left-predomiant, *BILATL* Symmetric neurodegeneration of the ATL

^*^Missing in 3 cases

^**^Missing in 4 cases

### Group assignment

Patients fulfilling all inclusion and no exclusion criteria were assigned to either of the three categories of patients with asymmetric right-predominant, left-predominant, or symmetric ATL involvement. The normal range of ATL metabolic asymmetry was defined based on the control group. Normalized regional glucose metabolism of the middle temporal pole region of the automated anatomical labeling atlas, version 3 (AAL3) [19], was read out, and an asymmetry index (AI) was calculated: AI = 200 × (right − left)/(right + left). Patients with an AI that fell within the 95% confidence interval were assigned to the group of patients with symmetric ATL neurodegeneration (“BILATL patients”). All other patients were assigned to the respective groups with asymmetric right- (“RATL patients”) or left-predominant neurodegeneration of the ATL (“LATL patients”).

### Statistics

Demographic variables were compared between patient and control groups by *t* tests for continuous or Fisher’s exact tests for categorical variables. The significance threshold for pairwise comparisons was set to *p* = 0.05.

Normalized regional FDG uptake was compared between groups by ANCOVA with factor “group,” controlling for PET scanner. Groups were contrasted in a pairwise fashion, such that contrast images depicted decreased FDG uptake in each of the patient groups compared to controls and each of the remaining patient groups (FWE-corrected *p* < 0.05; cluster extent *k* > 500 voxels of 1 mm isotropic size).

Cognitive performance of each of the patient groups (*z*-scores based on normative data) was tested for a difference from zero (i.e., from normative control data) by one-sample *t* tests (*p* < 0.05 after Bonferroni-correction for 9 test variables). Furthermore, cognitive performance was compared between patient groups by ANOVA and post-hoc Tukey HSD (*p* < 0.05 after Bonferroni-correction for 9 test variables).

We compared patients’ mortality risk between groups by a Cox proportional hazards model with 3-level-factor “group” and covariates “age” and “sex.” The significance threshold for pairwise comparisons was set to *p* = 0.05.

Furthermore, we searched clinical records for documented behavioral symptoms: four symptoms reported to be typical for right SD (social awkwardness, job loss, loss of insight, difficulties with person identification [2]) and the symptoms represented in the 12 items of the short version of the Neuropsychiatric Inventory (delusions, hallucinations, agitation/aggression, dysphoria/depression, anxiety, euphoria/elation, apathy/indifference, disinhibition, irritability/lability, aberrant motor behaviors, nighttime behavioral disturbances, and appetite/eating disturbances) [20]. We exploratively compared groups regarding the presence or absence of each of these symptoms using separate chi-square tests (significance threshold set to *p* = 0.05).

Group comparisons of PET images were performed with SPM12 and Matlab R2018b (The MathWorks, Inc.); all other computations were performed with R version 4.0.3 (www.R-project.org).

## Results

### Subjects

We included 35 patients in the analyses: 10 patients with right-, 17 with left-predominant, and 8 with symmetric neurodegeneration of the ATL. Amyloid PET was performed in 19/35 patients (negative according to clinical reports). Neither age nor sex differed between groups (*p* > 0.1, ANOVA and Fisher’s exact test, respectively). There were no significant differences between patient groups in symptom duration, education years, or MMSE (*p* > 0.1, ANOVA; Table 1). Information about handedness was available from 24/35 patients, of whom 23 were right-handed (7 patients with right-, 11 with left-predominant, and 5 with symmetric neurodegeneration of the ATL), one patient with symmetric ATL involvement was ambidextrous. Interestingly, two of the included patients were brothers, one with left-, the other with right-predominant ATL involvement. Please see Supplementary Fig. 1 for example of patient images.

### Metabolism

**Fig. 2:**
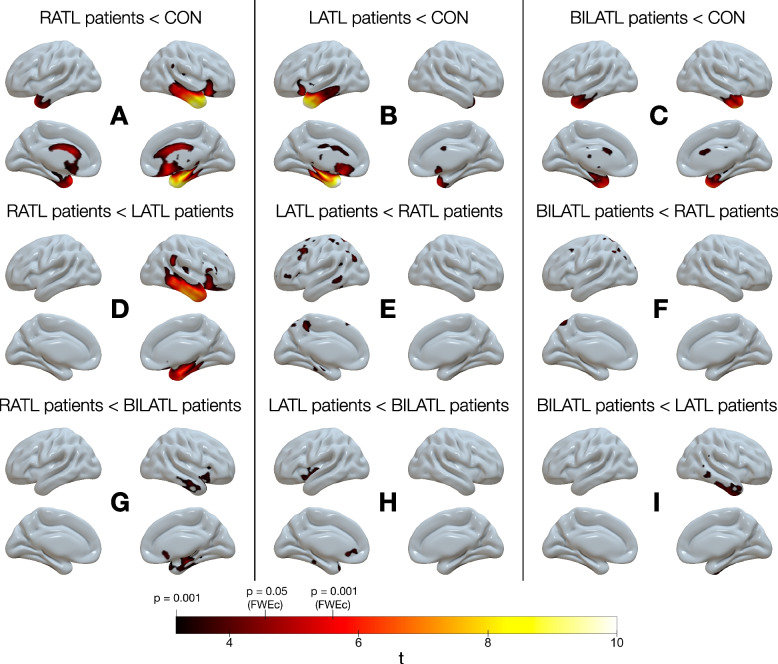
Surface projections of decreased FDG uptake in patients with right-predominant (RATL), left-predominant (LATL), or symmetric neurodegeneration of the ATL (BILATL) (uncorrected *p* < 0.001, *k* > 500). Please note that in the text, only results surviving voxel-level *p* < 0.05 (FWE-corrected) are reported and discussed. No other pairwise group comparison revealed significant results

Decreased normalized regional glucose metabolism was most extensive in patients with right-predominant neurodegeneration of the ATL (Fig. 2; please see also Supplementary Table 1). Compared to control subjects, it was observed in the right ATL, as expected, but also in the right orbitofrontal and anterior cingulate cortex. To a lesser extent, it was also decreased in the left ATL and left orbitofrontal cortex. Compared to patients with left-predominant ATL neurodegeneration, metabolism was decreased in the right ATL and right frontal and right parietal cortex. Compared to patients with symmetric ATL neurodegeneration, FDG uptake was reduced in two small clusters in the right inferior ATL and the right medial temporal lobe.

Patients with left-predominant ATL neurodegeneration showed significantly reduced FDG uptake when compared to controls in the left ATL and orbitofrontal cortex (Fig. 2).

The group with symmetric ATL neurodegeneration showed decreases compared to controls in both ATL and, compared to left-predominant patients, in a small cluster in the right inferior ATL (Fig. 2).

Neither the left-predominant nor the symmetric ATL neurodegeneration groups had significantly decreased metabolism compared to patients with right-predominant ATL neurodegeneration.

### Cognitive performance

**Fig. 3:**
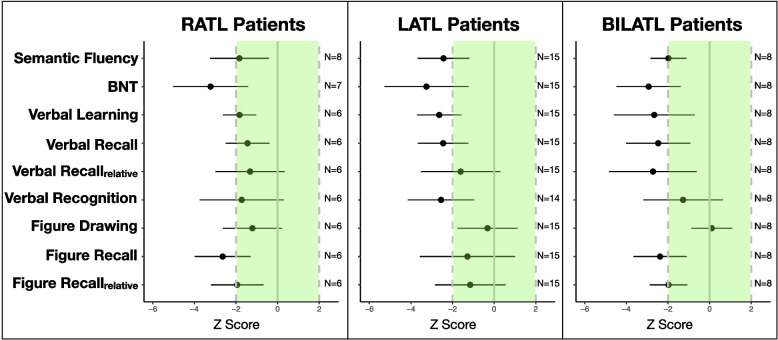
Cognitive performance (compared to normative data from the CERAD test battery, adjusted for age, sex, and education) in patients with right-predominant (RATL), left-predominant (LATL), and symmetric ATL neurodegeneration (BILATL). Performance was not significantly different between patient groups. Dots and error bars indicate mean ± one standard deviation. Light green area indicates mean ± 2 S.D. in controls (derived from CERAD-NAB normative data, controlled for age, sex, and education)

Subtest scores of the CERAD-NAB were available from 28 to 31 of the 35 patients. One-sample *t* tests revealed significantly impaired cognitive performance with reference to normative data (*p* < 0.05 after Bonferroni correction, Fig. 3): patients with right-predominant neurodegeneration of the ATL were impaired in naming, verbal learning, and figure recall. Patients with left-predominant neurodegeneration of the ATL showed deficits in naming, verbal learning, recall, recognition, and semantic fluency. Patients with symmetric ATL neurodegeneration were impaired in naming, verbal recall, figure recall (absolute and relative), and semantic fluency. None of the comparisons between patient groups reached significance, neither at Bonferroni-corrected nor uncorrected levels.

### Survival

**Fig. 4:**
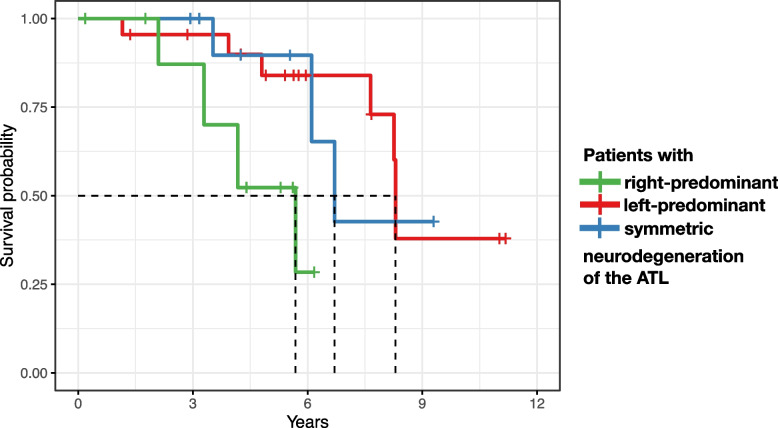
Age-adjusted mortality risk was higher in patients with right- than left-predominant ATL neurodegeneration (*p* < 0.05; age- and sex-adjusted Cox model). Dashed lines indicate median survival durations. *X*-axis depicts years after FDG PET

Thirteen of the 35 included patients died (4 with right-, 6 with left-predominant, 3 with symmetric ATL neurodegeneration) within the median follow-up duration of 7.7 years (95% C.I.: 5.5 to *undefined*). The age- and sex-adjusted Cox model (overall model fit: LR = 7.3 on 4 degrees of freedom, *p* = 0.12) revealed a significantly higher mortality risk in patients with right- compared to those with left-predominant ATL neurodegeneration (*p* = 0.042; HR = 6.1 [95% C.I. 1.1 to 34.7]). No other pairwise comparisons reached significance. Median survival durations were 5.7 for patients with right-, 8.3 years for patients with left-predominant, and 6.7 for patients with symmetric neurodegeneration of the ATL (Fig. 4).

We further performed an exploratory analysis of group effects on survival from the time of symptom onset, again using an age- and sex-adjusted Cox model. Survival duration from the time of symptom onset (available and analyzed in 31/35) did not differ between groups, particularly not between right- and left-predominant patients (*p* = 0.187; HR = 3.0 [95% C.I. 0.6 to 15.2]).

Documentation of behavioral symptoms was unavailable in 4/35 patients for most items (range 3 to 5 for all but the item “job loss,” where information was available for only 8/35 patients). Of the 35 patients, more than half had documented behavioral alterations regarding the items “loss of insight,” “agitation/aggression,” “dysphoria/depression,” “apathy/indifference,” and “nighttime behavioral disturbances.” There was no group difference in any of the items (all uncorrected *p* > 0.1).

## Discussion

Glucose metabolism differed between patient groups, with the greatest and most extensive decreases—involving also frontal cortical regions and the caudate nucleus—in patients with right-predominant neurodegeneration of the ATL. Profiles of cognitive deficits differed slightly between patient groups, but direct comparisons did not reach significance in any of the tests applied. Mortality risk was six times higher in patients with right- than those with left-predominant ATL neurodegeneration. Median survival was shortest in the right- and longest in the left-predominant group.

In order to be able to characterize the biological entity of right ATL neurodegeneration, we did not use a clinical syndrome (e.g., SD, svPPA, or bvFTD) as an inclusion criterion. However, a considerable proportion of our RATL and LATL patient groups had the clinical picture of rtvFTD and svPPA, respectively.

Hypometabolism in patients with right-predominant neurodegeneration of the ATL was not restricted to this region but also involved regions outside the temporal lobe, particularly in the right frontal lobe. This finding is consistent with previously reported frontal atrophy in rtvFTD [6] and in right SD [7]. This frontal involvement might be reflected by greater behavioral disturbances in right compared to left SD [3]. Although behavioral symptoms were present in all three groups in our study, their prevalence was not statistically different between groups. This might be due to the small number of patients and the retrospective search for documented symptoms in clinical records.

SvPPA is usually associated with TDP-C neuropathology [21]. The topography of neurodegeneration in our patient group with left-predominant ATL neurodegeneration was very similar to that of svPPA patients [6], that of patients with TDP-C [22, 23], and that of PPA patients with TDP-C [24]. Importantly, hypometabolism was overall more widespread in the right-predominant group, which might indicate that patients with right-predominant neurodegeneration of the ATL have been examined at a later disease stage.

All three patient groups were impaired in the naming task of the CERAD-NAB. Consistently, metabolism of the left ATL, the region most tightly linked to naming difficulties [25–28], was decreased in all three groups compared to controls.

All patient groups were impaired in at least one of the typical memory tests (wordlist learning and recall, figure recall). This unexpected finding might be due to our inclusion criteria: As noted above, in previous studies clinical syndromes and hence the presence of specific symptoms were often among the inclusion criteria. The majority of related previous studies included only patients fulfilling the criteria for SD which is per definition associated with semantic rather than episodic memory deficits [13]. In contrast, the current study included all patients with ATL neurodegeneration irrespective of the kind of cognitive symptoms.

Cognitive performance differed only slightly between patient groups, and differences did not reach statistical significance. The lack of differences between patients with right-predominant and those with left-predominant neurodegeneration of the ATL contradicts previous reports on patients with greater right versus left ATL involvement [1–6]. It might be explained by examination of patients with right-predominant ATL neurodegeneration at a later disease stage, with neurodegeneration already involving the contralateral temporal lobe. This could have led to similar impairment of the group with right-predominant ATL neurodegeneration in naming and verbal memory, functions usually assigned to the left hemisphere.

The only impaired “right-hemisphere” function of the right-predominant group was figure recall, reflecting that the standard test battery used here does not contain the most sensitive tests to detect right ATL degeneration (e.g., recognition of famous faces was not tested).

Survival durations were 5.7 to 8.3 years (patients with right- and left-predominant ATL neurodegeneration, respectively) after PET imaging. From clinical experience, it is our impression that PET imaging is usually performed close to the time of diagnosis, and hence, we compare the survival durations of our study to the literature that reported survival durations from the time of diagnosis. Importantly, whereas the median survival duration of patients with left-predominant ATL neurodegeneration fits previous results from studies in SD (8 to 10 years [12]) and svPPA (9 years [9]), median survival of the right-predominant group was closer to previous figures from bvFTD (4 years [8]; 5 years [9]). Therefore, patients with right-predominant neurodegeneration of the ATL have in common with bvFTD not only frontal neurodegeneration (see Fig. 2), but also a particularly short survival. The observed shorter survival of the right-predominant ATL group might be due to a faster progressing underlying disease. While it might be assumed that right ATL neurodegeneration is most often due to TDP-C neuropathology, as is left ATL neurodegeneration (and svPPA) [21], Josephs et al. reported tauopathies among patients with rtvFTD, particularly in patients whose atrophy pattern also involved the frontal lobe [29].

Mesulam and colleagues recently reported that among PPA patients, those with underlying TDP-A have a shorter survival after symptom onset than those with TDP-C neuropathology (median 6 vs 14 years, respectively) [24]. In our patient sample, neuropathology and hence the proportion of TDP-A cases are unknown. However, the reported atrophy pattern of TDP-A, with equal temporal, frontal, and parietal involvement [24], more closely resembled that associated with Alzheimer’s disease than TDP-C. Patients with such a pattern of neurodegeneration (on FDG PET) were not included in our study (see inclusion criterion 2).

An additional survival analysis of our data with survival duration computed *from estimated time of symptom onset* yielded no significant group difference. The observed shorter survival from the time of FDG PET but not from the time of symptom onset might indicate that patients with right-predominant were admitted to FDG PET later in the disease course than those with left-predominant ATL neurodegeneration.

Taken together, greater hypometabolism, similar cognitive impairment, and shorter survival in our view indicate that patients with right-predominant neurodegeneration of the ATL were examined at a later disease stage. Although symptom durations (from patient records) in our study did not statistically differ between patient groups, it has to be noted that symptom durations have only been estimated retrospectively at the time of clinical visit by patients and/or caregivers, which might bear considerable inaccuracy. As discussed by Chan et al. [5], the (initially) rather non-verbal nature of symptoms in patients with rtvFTD (and right-predominant ATL neurodegeneration) might lead to a delayed recognition of symptoms, which is in line with a report on later recognition and delayed therapy of right hemisphere compared to left hemisphere stroke [30]. Similarly, it has been shown that among patients with syndromes associated with frontotemporal lobar degeneration, those with svPPA receive the diagnosis of a neurodegenerative syndrome much earlier than all others, e.g., bvFTD patients [9].

### Limitations

Sample sizes were small in this retrospective, mono-centric study. Owing to the long time span during which FDG PET were acquired, different PET scanners were used. We tried to cope with this with different smoothing filters in image preprocessing and by including PET scanner as a covariate in statistical analyses. Patient selection in this study was based on imaging findings. It was neither based on clinical diagnoses, nor based on neuropathological findings (although all available amyloid PET were negative). It is hence possible that patients with non-neurodegenerative etiologies have been included. From all data presented here, we assume, however, that most included patients fall in the clinical spectrum of FTD and have a TDP-43 neuropathology, most likely TDP-C.

## Conclusions

Mortality risk was higher and neurodegeneration was more extensive in right than left ATL patients. This might indicate that right ATL patients consult memory clinics at a later disease stage than left ATL patients, when symptoms like naming and episodic memory deficits have already emerged. At time of diagnosis, the shorter survival duration of patients with right than left ATL neurodegeneration should be kept in mind when counseling patients and caregivers.

## Supplementary Information

**Additional file 1: Supplementary Figure 1.** NEUROSTAT 3D-SSP images of example patients with high, intermediate, and low certainty of the presence of the target hypometabolism (upper row: metabolism, lower row: hypometabolism; surface renderings from left, bottom, and right). **Supplementary Table 1.** SPM results of group comparisons (ANCOVA with PET scanner as covariate), thresholded at voxel-level *p* < 0.001 (uncorrected) and cluster extent k > 500 voxels of 1 mm isotropic size.

## Data Availability

The data that support the findings of this study are available on reasonable request from the corresponding author. The data are not publicly available as they contain information that could compromise the privacy of research participants.
