# Supplementary material for: More extensive hypometabolism and higher mortality risk in patients with right- than left-predominant neurodegeneration of the anterior temporal lobe
Source: Alzheimers Res Ther. 2023 Jan 10;15:11. doi: 10.1186/s13195-022-01146-w (PMC9830748; doi:10.1186/s13195-022-01146-w)
Supplement: Supplementary file 1 — Additional file 1: Supplementary Figure 1. NEUROSTAT 3D-SSP images of example patients with high, intermediate, and low certainty of the presence of the target hypometabolism (upper row: metabolism, lower row: hypometabolism; surface renderings from left, bottom, and right). Supplementary Table 1. SPM results of group comparisons (ANCOVA with PET scanner as covariate), thresholded at voxel-level p < 0.001 (uncorrected) and cluster extent k > 500 voxels of 1 mm isotropic size. [file 13195_2022_1146_MOESM1_ESM.docx]

Supplementary Figure 1. NEUROSTAT 3D-SSP images of example patients with high, intermediate, and low certainty of the presence of the target hypometabolism (upper row: metabolism, lower row: hypometabolism; surface renderings from left, bottom, and right).

The target hypometabolism, a metabolic pattern for svPPA or SD, was defined as predominant hypometabolism of the left, right, or both ATL. This hypometabolism may extend to frontal and (inferior) parietal cortices, but with a clear-cut gradient from the temporal pole to other regions and without clearly suggesting other neurodegenerative syndromes or diseases (e.g., Alzheimer’s disease, behavioral variant FTD, dementia with Lewy bodies, or atypical parkinsonian syndromes). Group assignment (RATL, LATL, BILATL patients) was based on asymmetry of normalized regional glucose metabolism of the middle temporal pole region from the AAL3 (please see Methods for details).

| **Supplementary Table 1.** SPM results of group comparisons (ANCOVA with PET scanner as covariate), thresholded at voxel-level p < 0.001 (uncorrected) and cluster extent k > 500 voxels of 1 mm isotropic size. Please note that in the main text, only clusters of voxels thresholded at a more rigid p < 0.05 (FWE-corrected) are reported and discussed. | | | | | | | | |
| --- | --- | --- | --- | --- | --- | --- | --- | --- |
| **Cluster p Value (FWE-corrected)** | **Cluster Size (Number of Voxels)** | **Cluster p Value (uncorrected)** | **Peak Voxel p Value (FWE-corrected)** | **Peak Voxel t Value** | **Peak Voxel p Value (uncorrected)** | **x {mm}** | **y {mm}** | **z {mm}** |
| **RATL patients < CON** | | | | | | | | |
| 3.9968E-15 | 127714 | 1.38136E-15 | <1E-16 | 10.40187931 | 4.44089E-16 | 52 | -4 | -40 |
| 5.80711E-05 | 22742 | 1.98013E-05 | 1.59721E-05 | 6.585057259 | 8.27092E-10 | -40 | 20 | -33 |
| 0.457645643 | 1252 | 0.208619755 | 0.019102793 | 4.82688427 | 2.2727E-06 | 8 | 66 | 23 |
| **RATL patients < LATL patients** | | | | | | | | |
| 2.84783E-12 | 92830 | 9.7103E-13 | 8.17316E-10 | 8.695807457 | 2.0095E-14 | 65 | -27 | -23 |
| 8.73214E-06 | 28964 | 2.97744E-06 | 0.000120734 | 6.116836548 | 7.61978E-09 | 59 | 20 | 14 |
| 0.419939956 | 1394 | 0.185702231 | 0.030140229 | 4.697042465 | 3.87048E-06 | 11 | 54 | 41 |
| **RATL patients < BILATL patients** | | | | | | | | |
| 1.95888E-07 | 42853 | 6.67927E-08 | 0.001360725 | 5.526574612 | 1.12524E-07 | 62 | -16 | -34 |
| 0.125488131 | 3458 | 0.045720936 | 0.079984938 | 4.404400826 | 1.24456E-05 | 8 | 20 | -7 |
| **LATL patients < CON** | | | | | | | | |
| 1.31642E-08 | 53776 | 4.48864E-09 | <1E-16 | 11.35635281 | 4.44089E-16 | -31 | 14 | -39 |
| 0.010361607 | 8548 | 0.003551465 | 2.18273E-06 | 7.029669285 | 9.47502E-11 | 29 | 16 | -41 |
| 0.001448248 | 13403 | 0.000494172 | 5.13198E-05 | 6.317183495 | 2.97117E-09 | -26 | 12 | -22 |
| 0.655111001 | 651 | 0.36297742 | 0.009149104 | 5.029773712 | 9.72836E-07 | 4 | 12 | -16 |
| 0.679057255 | 589 | 0.387513831 | 0.071834175 | 4.437871933 | 1.09145E-05 | 9 | 11 | 6 |
| 0.176342179 | 2856 | 0.066148892 | 0.413433553 | 3.810185194 | 0.000115086 | -4 | 7 | 27 |
| **LATL patients < RATL patients** | | | | | | | | |
| 0.018120072 | 7301 | 0.006235127 | 0.000349942 | 5.862127781 | 2.47396E-08 | -68 | -43 | 3 |
| 0.383246961 | 1545 | 0.16478792 | 0.006888931 | 5.106101513 | 7.03418E-07 | -39 | 22 | 8 |
| 0.483821863 | 1160 | 0.225486913 | 0.016229622 | 4.872467041 | 1.8815E-06 | -51 | 41 | 2 |
| 0.055739968 | 4980 | 0.019556089 | 0.023452783 | 4.768914223 | 2.88556E-06 | -47 | 13 | 42 |
| 0.467721232 | 1216 | 0.215013769 | 0.047579897 | 4.563058853 | 6.64421E-06 | -21 | -58 | 66 |
| 0.242522467 | 2309 | 0.094709276 | 0.051002058 | 4.542265892 | 7.21935E-06 | -8 | -43 | 50 |
| 0.618959313 | 748 | 0.328987976 | 0.087136619 | 4.377457142 | 1.38267E-05 | -6 | -70 | 57 |
| 0.705642818 | 522 | 0.416997403 | 0.131326852 | 4.244467258 | 2.31063E-05 | 17 | -79 | 2 |
| 0.692061541 | 556 | 0.401617447 | 0.193159467 | 4.111708641 | 3.81958E-05 | -25 | -54 | 40 |
| 0.309772015 | 1898 | 0.126410236 | 0.276848099 | 3.977920532 | 6.27307E-05 | -34 | -29 | -27 |
| 0.658929716 | 641 | 0.366773837 | 0.495753396 | 3.723341942 | 0.000156499 | -51 | -48 | 22 |
| 0.468287253 | 1214 | 0.215376551 | 0.530267099 | 3.688480616 | 0.000176815 | -8 | 26 | 54 |
| **LATL patients < BILATL patients** | | | | | | | | |
| 0.048446075 | 5256 | 0.016932376 | 0.125584268 | 4.259318829 | 2.18293E-05 | -39 | 1 | -10 |
| 0.38580663 | 1534 | 0.166205984 | 0.131784907 | 4.243306637 | 2.32091E-05 | -5 | 27 | -7 |
| 0.663919269 | 628 | 0.371798821 | 0.206230257 | 4.088210106 | 4.17053E-05 | -30 | -31 | -24 |
| **BILATL patients < CON** | | | | | | | | |
| 1.05771E-05 | 28312 | 3.60653E-06 | 3.07973E-08 | 7.943881989 | 9.58567E-13 | 29 | 14 | -43 |
| 1.38764E-05 | 27397 | 4.73153E-06 | 9.23501E-06 | 6.708852291 | 4.54818E-10 | -29 | 9 | -42 |
| 0.229727884 | 2401 | 0.088997979 | 0.004972402 | 5.192675591 | 4.85375E-07 | -9 | 14 | 9 |
| 0.234093581 | 2369 | 0.090936023 | 0.016663341 | 4.86511898 | 1.93981E-06 | 11 | 11 | 13 |
| 0.400805316 | 1471 | 0.174635958 | 0.016961473 | 4.860172272 | 1.98005E-06 | -29 | -32 | 17 |
| 0.232172662 | 2383 | 0.090081921 | 0.240791256 | 4.031209946 | 5.15477E-05 | 7 | 22 | 19 |
| 0.70483886 | 524 | 0.416067395 | 0.282235392 | 3.970393419 | 6.44859E-05 | 31 | -33 | 19 |
| **BILATL patients < RATL patients** | | | | | | | | |
| 0.015005579 | 7715 | 0.005155281 | 0.016842673 | 4.862133503 | 1.964E-06 | -33 | -73 | 50 |
| 0.644885379 | 678 | 0.35301486 | 0.051957186 | 4.536691189 | 7.38155E-06 | -47 | 13 | 43 |
| 0.566976411 | 896 | 0.285382224 | 0.061441954 | 4.485907078 | 9.03072E-06 | -46 | -60 | 48 |
| **BILATL patients < LATL patients** | | | | | | | | |
| 0.001250897 | 13792 | 0.00042679 | 0.004706826 | 5.207139969 | 4.5605E-07 | 69 | -19 | -16 |
